# Defining a Conformational Consensus Motif in Cotransin-Sensitive Signal Sequences: A Proteomic and Site-Directed Mutagenesis Study

**DOI:** 10.1371/journal.pone.0120886

**Published:** 2015-03-25

**Authors:** Wolfgang Klein, Carolin Westendorf, Antje Schmidt, Mercè Conill-Cortés, Claudia Rutz, Marcus Blohs, Michael Beyermann, Jonas Protze, Gerd Krause, Eberhard Krause, Ralf Schülein

**Affiliations:** Leibniz-Institut für Molekulare Pharmakologie (FMP), Robert-Rössle-Str. 10, 13125, Berlin, Germany; Yong Loo Lin School of Medicine, National University of Singapore, SINGAPORE

## Abstract

The cyclodepsipeptide cotransin was described to inhibit the biosynthesis of a small subset of proteins by a signal sequence-discriminatory mechanism at the Sec61 protein-conducting channel. However, it was not clear how selective cotransin is, i.e. how many proteins are sensitive. Moreover, a consensus motif in signal sequences mediating cotransin sensitivity has yet not been described. To address these questions, we performed a proteomic study using cotransin-treated human hepatocellular carcinoma cells and the stable isotope labelling by amino acids in cell culture technique in combination with quantitative mass spectrometry. We used a saturating concentration of cotransin (30 micromolar) to identify also less-sensitive proteins and to discriminate the latter from completely resistant proteins. We found that the biosynthesis of almost all secreted proteins was cotransin-sensitive under these conditions. In contrast, biosynthesis of the majority of the integral membrane proteins was cotransin-resistant. Cotransin sensitivity of signal sequences was neither related to their length nor to their hydrophobicity. Instead, in the case of signal anchor sequences, we identified for the first time a conformational consensus motif mediating cotransin sensitivity.

## Introduction

Signal sequences of secretory and integral membrane proteins are mediators of the early steps of protein biogenesis and transport in cells [1–3]. After their synthesis at cytosolic ribosomes, signal sequences bind the signal recognition particle (SRP) and initiate targeting of the ribosome/nascent chain/SRP complex to the SRP receptor of the translocon machinery at the endoplasmic reticulum (ER) membrane. Signal sequences are also involved in translocon gating. They bind to the cytosolic side of the protein-conducting Sec61 channel and destabilize its closed conformation. Secretory proteins possess invariantly signal sequences which are located at the N-terminus of the proteins, the so-called signal peptides (SP). SPs are usually cleaved off following translocation of the proteins across the ER membrane. The signal sequences of integral membrane proteins do not mediate transfer across the ER membrane but integration of the proteins into the bilayer. Integral membrane proteins may also possess SPs. The majority, however, contain so-called signal anchor sequences (SAS) which are uncleaved and form part of the mature protein (usually the first transmembrane domain, TM1).

Cotransin is a derivative of the fungal substance HUN-7293 [4, 5]. Like Hun-7293, cotransin was shown to inhibit the Sec61 protein-conducting channel of the translocon complex in the presence of specific SPs [4, 5]. As a consequence, the cotranslational translocation of the target proteins is prevented in a SP discriminatory mechanism of action. Originally, only a small subset of proteins was reported to possess cotransin-sensitive SPs and it was suggested that cotransin is a rather selective substance. The originally described group of cotransin-sensitive proteins is formed by vascular cell adhesion molecule 1 (VCAM1), P-selectin, angiotensinogen, β-lactamase and the G protein-coupled corticotropin-releasing factor receptor type 1, an integral membrane protein [4]. Recently, another G protein-coupled receptor, namely the endothelin B receptor was shown to be cotransin-sensitive [6]. Interestingly, no SAS-containing protein was found among this original subset of proteins. However, recent results showed that at least one SAS, namely that of the tumor necrosis factor alpha (TNF-α) is also cotransin-sensitive [7, 8].

The detailed mechanism of action of cotransin or the other cyclodepsipeptides is still not completely clear. To date, it was shown that cotransin neither affects SRP binding nor targeting of the ribosome/nascent chain/SRP complex to the ER membrane [4]. Crosslinking experiments suggested that the substances interact with the Sec61α subunit (protein-conducting channel) of the translocon complex [5] and dislocate the sensitive nascent chains to the Sec61β subunit [5, 9]. These data suggest that the cyclodepsipeptides may compete with the sensitive SPs for binding to a specific acceptor site within the Sec61α subunit [10].

To date, a consensus motif mediating cotransin sensitivity was not described although some critical residues were identified in sensitive signal sequences. [7, 9, 10]. Moreover, it is not known how selective cotransin is, i.e. how many proteins are indeed sensitive and whether SASs may also be affected in significant amounts.

To address all these questions, we performed a proteomic study and analyzed the expression of secreted and integral membrane proteins of the human hepatocellular liver carcinoma cell line (HepG2) following cotransin treatment at saturating concentrations (30 μM). Sensitive proteins were identified using the stable isotope labelling by amino acids in cell culture technique (SILAC) in combination with quantitative mass spectrometry.

## Materials and Methods

### Material

The aquaporin 2 protein (AQP2) cDNA was kindly provided by Enno Klußmann (Berlin, Germany. The vector plasmids pEGFP-C1 and pEGFP-N1 and the ER marker plasmid pECFP-ER were from Clontech (Mountain View, Ca, USA). The QuickChange site-directed mutagenesis kit was from Stratagene (Heidelberg, Germany). The plasma membrane stain trypan blue and the transfection reagent polyethylenimine (PEI) were from Merck-Millipore (Darmstadt, Germany). Cotransin was synthesized in our group using our previously described solid phase protocol (purity 95%; no TFA/ acetic salt) [11–13] and dissolved it in dimethyl sulfoxide (DMSO). [^125^I]ET-1 (2000 Ci/mmol) was purchased from Amersham Biosciences (Freiburg, Germany). The human embryonic kidney 293T (HEK 293) cells were from Clontech Laboratories, Inc. (Mountain View, CA, USA), the HepG2 cells were a gift of G. Püschel (Potsdam, Germany). The RotiLoad sample buffer was from Carl Roth (Karlsruhe, Germany).

Monoclonal antibodies (dilution for immunoblots in brackets): the anti-apolipoprotein B-100 (Apo B-100) antibody was purchased from Santa Cruz Biotechnology (Dallas, TX, USA; No. sc-13538, 1:300), the anti-GFP antibody was from Clontech Laboratories (Heidelberg, Germany; No. JL-8, 1:4,000), the anti-tubulin antibody was from Calbiochem (Billerica, MA, USA; No. CP06, 1:1,000).

Polyclonal antibodies (all rabbit, dilution for immunoblots in brackets): the anti-cadherin-2 (CDH2) antibody was purchased from Sigma-Aldrich (Taufkirchen, Germany; No. C3678, 1:1,000), the anti-calnexin (CNX) antibody was from Stressgen (Victoria, Canada; No. SPA 860, 1:1,000), the anti-claudin-1 (CLDN1) antibody was from Invitrogen (Carlsbad, CA, USA; No. 71–7800, 1:2,000), the anti-plasminogen-activator inhibitor 1 (PAI-1) antibody was from Millipore (Billerica, MA, USA; No. 09–726, 1:1,000), the anti-glyceraldehyde-3-phosphate dehydrogenase (GAPDH) antibody (No. 14C10, 1:1,000) and the anti-erlin-2 antibody (No. 2959S, 1:500) were from Cell Signaling Technology (Danvers, MA, USA).

Secondary antibodies (dilution for immunoblots in brackets): peroxidase-conjugated AffiniPure goat anti-mouse IgG (1:2,500), peroxidase-conjugated AffiniPure goat anti-rabbit IgG (1:5,000) and alkaline phosphatase-conjugated AffiniPure goat anti-mouse IgG (1:1,500) were purchased from Dianova (Hamburg, Germany). Capillary columns for LC separations (PepMap100, C18, 3 μm, 100 Å, 250 mm × 75 μm i.d.) were from Thermo Fisher Scientific (Waltham, MA, USA). All other reagents were from Sigma-Aldrich (Munich, Germany).

### Cell culture

HepG2 cells and HEK 293 cells were cultured at 37°C and 5% CO_2_ in Dulbecco’s modified Eagle’s medium (DMEM, low glucose, GlutaMAX) containing 10% (v/v) fetal calf serum (FCS), penicillin (100 U/ml) and streptomycin (100 μg/ml).

### Plasmid constructions and site-directed mutagenesis

Standard DNA manipulations were carried out. The AQP2 cDNA was cloned into a the vector plasmid pEGFP-N1 thereby replacing the stop codon of AQP2. The resulting fusion construct WT.AQP2 encodes AQP2 C-terminally tagged with GFP. Introduction of the putative conformational consensus motif into the SAS of AQP2 (combined point mutations F25G, F26G, G27L, Q33K) was carried out by site-directed mutagenesis using the QuickChange site-directed mutagenesis kit from Stratagene (Heidelberg, Germany) according to the supplier’s recommendations. The resulting mutant was CM.AQP2. The truncated construct WT.AQP2.NT encodes an N-terminal EGFP fusion to an AQP2 fragment (amino acid residues 1–40 of AQP2) consisting of the N terminus, TM1 and the first extracellular loop in the pEGFP-C1 vector from Clontech. Mutant CM.AQP2.NT (combined point mutations F25G, F26G, G27L, Q33K) was derived by site-directed mutagenesis as described above. The nucleotide sequences of all plasmid constructs were verified by sequencing (Source Biosciences Lifesciences, Berlin, Germany).

### SILAC methodology

SILAC experiments [14–16] were carried out according to the supplier's recommendations outlined in the Pierce SILAC protein quantification kits. To obtain 90% labelling, HepG2 cells were grown on 60 mm diam. dishes for 2 weeks in DMEM containing either ^12^C_6_ L-Lysine (1 mM) and ^12^C_6_^14^N_4_ L-Arginine (0.48 mM) (“light” sample) or ^13^C_6_ L-Lysine (0.96 mM) and ^13^C_6_^15^N_4_ L-Arginine (0.45 mM) (“heavy” sample). Cells of the light sample were treated 17 h with 30 μM of cotransin whereas cells of the heavy sample served as a DMSO-treated control. The cell culture media of the light and heavy samples were removed and combined for the isolation of the secretory proteins (see below).

For the isolation of the total integral membrane proteins, cells were washed twice with cold PBSI [phosphate buffered saline (PBS) containing 0.5 mM phenylmethylsulfonylfluorid (PMSF), 3.2 μg/μl soybean trypsin inhibitor (STI), 0.5 mM benzamidine, 1.4 μg/μl trasylol; pH 7.4]. Cells were disrupted in 100 μl cold fractionation buffer [250 mM sucrose, 20 mM 4-(2-hydroxyethyl)-1-piperazineethanesulfonic acid, 10 mM KCl, 1.5 mM MgCl_2_, 1 mM ethylenediamine tetra-acetate (EDTA), 1 mM ethylene glycol tetraacetic acid (EGTA), 0.5 mM PMSF, 3.2 μg/μl STI, 0.5 mM benzamidine, 1.4 μg/μl trasylol; pH 7.4] using a potter homogenizer. Lysates of the light and heavy samples were combined and cell nuclei were removed by centrifugation (4°C, 720 x g, 5 min). The plasma membrane-containing fraction was collected (4°C, 100,000 x g, 1 h) resuspended in fractionation buffer and centrifuged for a second time as described above. The pellet containing the integral membrane proteins was resuspended in 50 μl Rotiload buffer supplemented with 15 μl Tris-HCl (100 mM, pH 8.5). Proteins were reduced using dithiothreitol (DTT) (5 mM, 30 min, 55°C) and alkylated using iodoacetamide (15 mM, 30 min, room temperature) in the dark. Separation of the proteins by sodium dodecyl sulfate polyacrylamide gel electrophoresis (SDS-PAGE) was carried out as described previously [6].

Total secreted proteins from the combined cell culture media of the light and heavy sample (see above) were precipitated by adding 1 volume of 100% trichloroacetic acid (TCA) to 4 volumes of the cell culture medium and incubated at 4°C for 10 min. After centrifugation (13,000 x g, 5 min), proteins were washed 3 times with 200 μl cold acetone. The proteins were resuspended, reduced and alkylated for SDS-PAGE in Rotiload sample buffer as described above for the total membrane proteins.

All experiments for total secretory and membrane proteins were repeated with switched isotopic coding (forward and reverse experiment).

### Protein identification by mass spectrometry

Tryptic digest of proteins following SDS-PAGE and nano liquid chromatography—mass spectrometry/mass spectrometry (LC-MS/MS) experiments were essentially done as described [17]. In brief, gel slices were washed with 50% (v/v) acetonitrile in 50 mM ammonium bicarbonate, dehydrated in acetonitrile and dried in a vacuum centrifuge. The dried gel pieces were reswollen in 20 μl of 50 mM ammoniumbicarbonate containing 50 ng of trypsin (sequencing-grade). After overnight incubation at 37°C, 15 μl of 0.3% TCA in acetonitrile was added and the separated supernatant was vacuum-dried. Prior to mass spectrometry (MS) analysis, the peptides were dissolved in 6 μl of 0.1% TCA and 5% acetonitrile in water.

Liquid chromatography (LC) separations were performed on a capillary column (PepMap100, C18, 3 μm, 100 Å, 250 mm × 75 μm i.d.) at an eluent flow rate of 200 nl/min using a gradient of 3–50% mobile phase B in 90 min. Mobile phase A contained 0.1% formic acid in water and mobile phase B contained 0.1% formic acid in acetonitrile. Mass spectra were acquired in a data-dependent mode with one MS survey scan (with a resolution of 30,000) in the Orbitrap and MS/MS scans of the four most intense precursor ions in the linear trap quadrupole.

Identification and quantification of proteins were carried out with version 1.0.13.13 of the MaxQuant software package as described [18]. Data were searched against the international protein index human protein database (version 3.52). The mass tolerance of precursor and sequence ions was set to 7 ppm and 0.35 Da, respectively. Methionine oxidation and the acrylamide modification of cysteine were used as variable modifications. False discovery rates were <1% based on matches to reversed sequences in the concatenated target-decoy database.

### Bioinformatic tools

For the hydrophobicity analysis, the grand averages of hydropathicity (GRAVY) values were determined for the signal sequences using the GRAVY calculator software (S. Fuchs, University of Greifswald, Greifswald, Germany). The frequency of signal sequence hydrophobicity (sorted in classes of hydrophobicity ranging from 0–100 in steps of 4 in a scale of 0–25) was plotted against total hydrophobicity of the signal sequences (ranging from 0–100). For the signal sequence length analysis, the frequency of signal sequence length (sorted in classes ranging from 0–50 amino acid residues in steps of 4 in a scale from 0–100) was plotted against total length of the signal sequences.

The signal sequence alignments were prepared using the ClustalW software (European Bioinformatics Institute, EBI, Cambridge, UK) and manual refinements. The conformational consensus motif in the signal sequences was identified using the *fuzzpro* application of the EMBOSS calculator and the Geneious Pro software *5*.*4*.*4* (available from http://www.geneious.com) [19]. The same software was used for the motif screen.

Helical structure prediction and surface visualization of the signal sequences were carried out using the PyMol software package (Schrödinger Inc., Cambridge, MA, USA).

### Detection of cotransin-sensitive secreted and integral membrane proteins by immunoblotting

HepG2 cells (2 x 10^6^) were grown in a 60 mm diam. dishes for 24 h. Cells were washed twice with phosphate-buffered saline (PBS; pH 7.4), incubated in serum free medium for 3 h, washed again and cultured in serum free medium containing cotransin (30 μM) for another 17 h. Total secreted and integral membrane proteins were isolated using the cell fractionation protocol (see above) and resuspended in Roti-Load sample buffer. Proteins were separated on a SDS gradient gel (5–12%, 20 mA, proteins from 5 x 10^5^ cells/lane) and detected by immunoblotting [20] using the monoclonal or polyclonal antibodies against the target proteins and peroxidase-conjugated anti-mouse or anti-rabbit IgG respectively (see the Material paragraph for the antibodies and dilutions). Blocking of unspecific interactions was carried out using 5% skim milk powder in PBS. The intensities of the protein bands were quantified densitometrically using the NIH image analysis software (ImageJ Version 1.48, National Institutes of Health, Bethesda, MD, USA)

### Confocal laser scanning microscopy (LSM)

(A) Colocalization of WT.AQP2.NT or CM.AQP2.NT and the ER marker ECFP-ER. HEK 293 cells (3.0 × 10^5^) were grown for 24 h on 30 mm glass coverslips in 35 mm diam. dishes. Cells were transiently co-transfected with 0.8 μg of plasmid DNA of WT.AQP2.NT or CM.AQP2.NT and 0.8 μg of ECFP-ER using PEI according to the supplier’s recommendations. After another 24 h of incubation, the coverslips were transferred into a self-made chamber (details on request) and covered with PBS without Ca^2+^ and Mg^2+^. Fluorescence signals were visualised using the laser scanning microscope system LSM710-ConfoCor3 (Carl Zeiss Microscopy GmbH, Jena, Germany, 63x/1.3 oil objective). The GFP fluorescence signals of WT.AQP2.NT or CM.AQP2.NT were detected on one channel (argon laser λ_exc_ = 488 nm, emission 491–603 nm band pass filter) and the CFP fluorescence signals of ECFP-ER on the second channel (argon laser λ_exc_ = 458 nm, emission 461–501 nm band pass filter) using a multi beam splitter MBS 488 (channel one) and a MBS 458 (channel two). The overlay of the signals was computed. Images were analyzed using the ZEN 2010 software (Carl Zeiss Microscopy GmbH, Jena, Germany).

(B) Colocalization of the soluble (unfused) GFP protein and the plasma membrane stain trypan blue. HEK 293 cells were transiently transfected with the vector plasmid pEGFP-C1 as described above. After 24 h of incubation, the trypan blue solution was added to the cells (final concentration 0.05%, w/v) and cells were incubated for 1 min. The GFP fluorescence signals were detected on one channel (argon laser λ_exc_ = 488 nm, emission 491–603 nm band pass filter) and trypan blue signals on the second channel (argon laser λ_exc_ = 561nm, emission 564–704 band pass filter) using a multi beam splitter MBS 488 (channel 1) and a MBS 561 (channel two). The overlay of the signals was computed. Images were analyzed using the ZEN 2010 software (Carl Zeiss Microscopy GmbH, Jena, Germany).

### Flow cytometry biosynthesis inhibition assay

HEK 293 cells (4.5 x 10^5^) grown on 12-well plates for 20 h were transiently transfected with 1.2 μg plasmid DNA and PEI per well according to the suppliers’s recommendations. Cells were incubated for 4.5 h and treated for 19 h with cotransin (final concentration 10 μM or 1–50 μM for a concentration-response curve) or cycloheximide (final concentration 0.1 μg/ml) or DMSO (negative control). Final DMSO concentration in all samples was 1.5%. Cells were washed twice with PBS and the GFP fluorescence signals of the constructs were analyzed by flow cytometry using a FACSCalibur system (BD Biosciences, USA). For each sample, total fluorescence intensity of 1 x 10^4^ cells was analysed using the BD CellQuest Pro software (BD Biosciences, USA). The total amount of GFP fluorescence was normalized by subtracting the background of non-transfected HEK 293 cells. To eliminate the portion of the GFP fluorescence present at time t_0_ of cotransin treatment (i.e proteins synthesized during the 4.5 h incubation time following transfection which may endure thereafter the cotransin incubation time), we subtracted the value of cycloheximide-treated cells. In the case of the concentration response curve, data of the cotransin-treated cells were normalized to the DMSO control (100%).

### Statistics

Unless otherwise indicated, analyses were performed using the Student’s t-test (GraphPad *t* test calculator, GraphPad Software, Inc., La Jolla, CA); *p* values < 0.001 were considered to be significant.

## Results

### Cotransin sensitivity of secretory and integral membrane proteins of HepG2 cells

**Fig 1:**
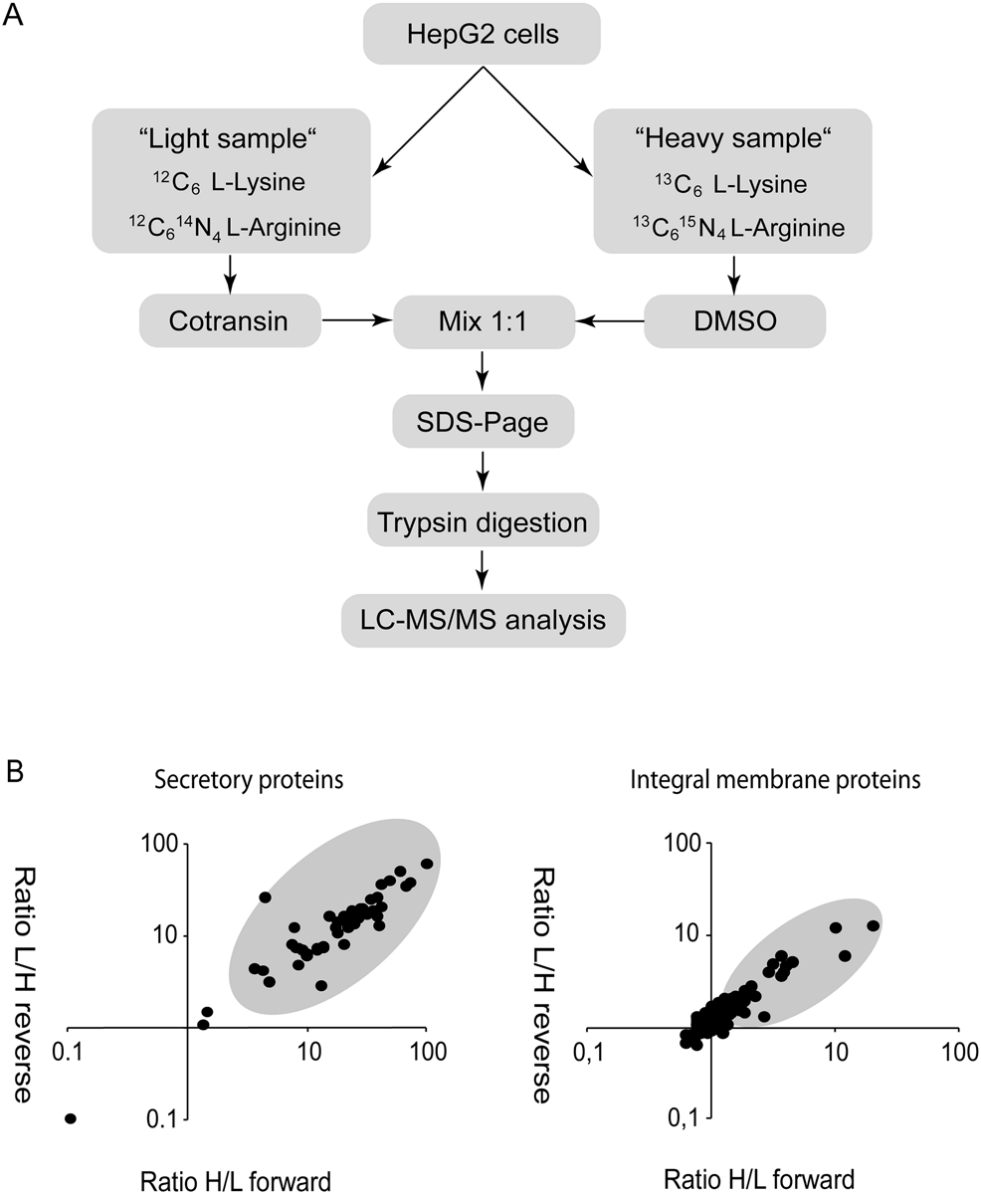
Strategy and results of the SILAC experiments. A. Schematic representation of the SILAC approach. Proteins of HepG2 cells were labelled with either ^12^C_6_ L-Lysine and ^12^C_6_^14^N_4_ L-Arginine (“light” sample) or ^13^C_6_ L-Lysine and ^13^C_6_^15^N_4_ L-Arginine (“heavy” sample). Cells were treated for 17 h with cotransin (30 μM) or DMSO and pooled cell lysates or supernatants containing total secretory or integral membrane proteins were separated using a SDS gradient gel (4–12%). The proteins bands were cut out, proteins were digested using trypsin and finally analysed using LC-MS/MS. The forward experiment is shown. For the reverse experiment isotopic coding was inverted. **B**. SILAC results. Shown are dotplots for secretory (left panel) and integral membrane proteins (right panel) which were identified and quantified by LC-MS/MS analysis (see also the S1 Table for a list of the individual proteins). The ratios of protein expression following DMSO or cotransin treatment of the forward (heavy/light sample = H/L) and reverse experiments (light/heavy sample = L/H) are plotted against each other. The grey areas indicate proteins which were considered to be inhibited by cotransin.

The SILAC approach used [14–16] is outlined in Fig. 1A. HepG2 hepatocytes were used because they are known to secrete a broad range of major plasma and other secretory proteins. Moreover, these cells are easy to wash because of their epithelial morphology. For the few sensitive proteins reported, the IC_50_ values for cotransin-mediated biosynthesis inhibition were in a range of 0.5–5 μM [4, 6]. To identify less-sensitive proteins and to discriminate the latter from completely resistant proteins, we used a cotransin concentration of 30 μM for our study which is a saturating concentration taking the reported IC_50_ values into account. It was previously demonstrated that cotransin does not affect transcription [4] and does not cause cytotoxicity at a concentration of 30 μM [6]. This may be explained by the fact that cotransin treatment usually does not lead to a complete inhibition of the biosynthesis of the sensitive proteins.

Cells were grown in medium either containing ^12^C_6_ L-Lysine and ^12^C_6_^14^N_4_ L-Arginine (“light” sample) or ^13^C_6_ L-Lysine and ^13^C_6_^15^N_4_ L-Arginine (“heavy” sample) (Fig. 1A). Cells of the light sample were treated with cotransin whereas cells of the heavy sample served as a DMSO-treated control. Total secreted proteins of both samples were mixed, isolated, and separated by SDS-PAGE. Proteins were in-gel digested with trypsin and the resulting peptides were subjected to LC-MS/MS analysis. For the analysis of the integral membrane proteins, labelling and cotransin or DMSO-treatment of the cells were performed accordingly. Total cell lysates of the light and heavy samples were mixed and cell fractionations were performed. Proteins of crude membrane preparations were separated by SDS-PAGE, in-gel digested and analysed using LC-MS/MS as described above for the secretory proteins.

All experiments were performed twice in a crossover mode, meaning that the light and heavy labelled SILAC samples were treated with cotransin and DMSO, respectively, and *vice versa*. In these experiments, we considered proteins as cotransin-sensitive if the ratio of protein expression following DMSO or cotransin treatment (DMSO/cotransin) was higher than 1.65 in the forward and reverse experiment.

A total of 217 proteins could be detected in substantial amounts in both experiments: 53 of them were secreted proteins and 164 integral membrane proteins (Fig. 1B; see the S1 Table for the complete dataset). Surprisingly, 50 out of the 53 secretory proteins (all SP) showed a significant decrease in protein expression following cotransin treatment. In contrast, only 21 of the integral membrane proteins were cotransin-sensitive (SP = 9; SAS = 11; signal sequence not specified = 1) whereas 143 were non-sensitive (SP = 47; SAS = 94; signal sequence not specified = 2). Thus, at this saturating concentration, cotransin does not discriminate between different secretory proteins which are all more or less sensitive. Instead, the substance discriminates mainly between secretory and integral membrane proteins.

### Integral membrane proteins possessing SASs may also be cotransin-sensitive

Secretory proteins possess invariantly cleavable SPs. In the case of integral membrane proteins, only a minority possesses SPs, the majority contains SASs. An obvious hypothesis to explain the fact that at saturating concentration almost all secretory but only a few integral membrane proteins were cotransin-sensitive is, that the substance might affect selectively SP-containing proteins. However, among the 21 sensitive integral membrane proteins found, 9 possess SPs and 11 SASs (see the S1 Table). These results show that at least in the case of the sensitive integral membrane proteins identified in our study, SPs and SASs seem to be affected without preference.

### Confirmation of the SILAC results by expression analysis of specific proteins

**Fig 2:**
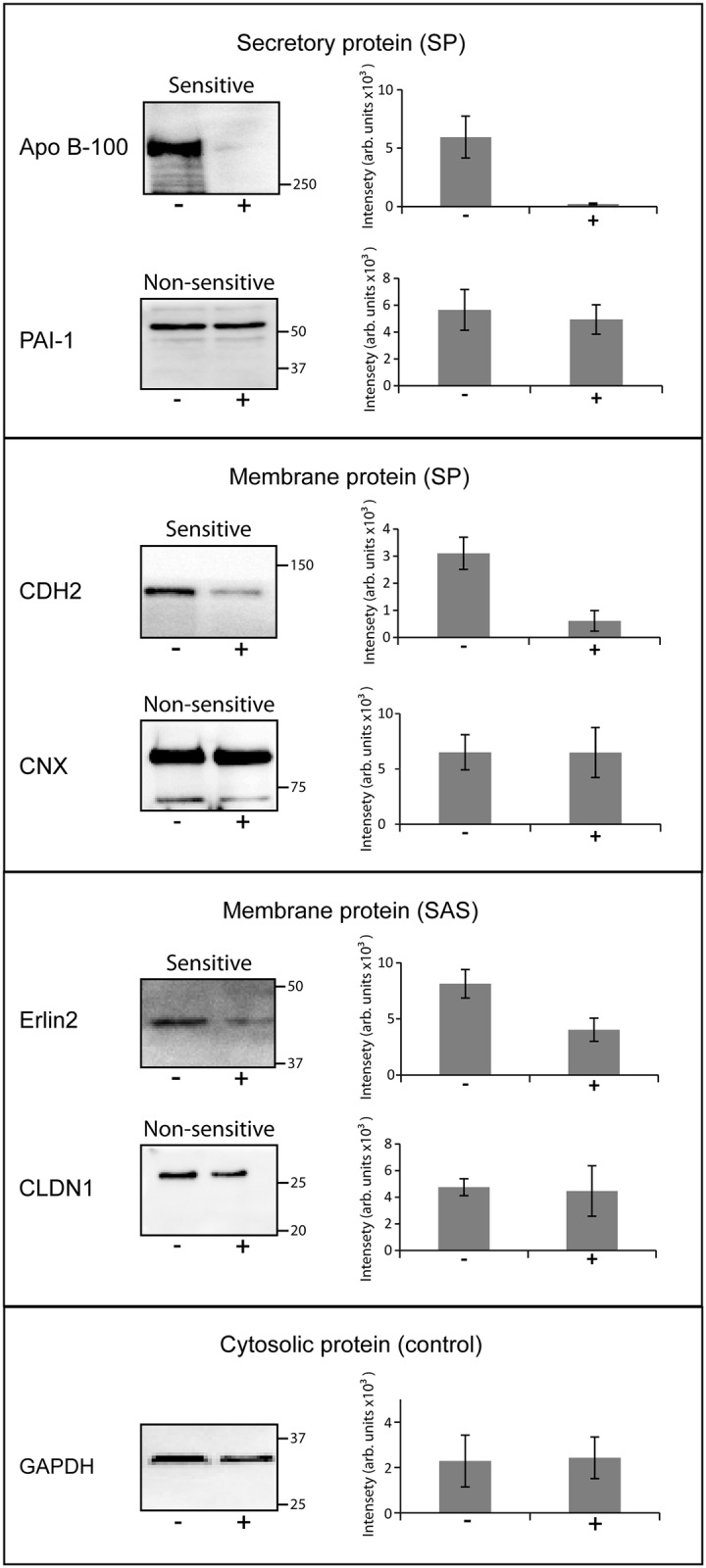
Confirmation of cotransin sensitivity and cotransin resistance of selected proteins using SDS-PAGE/ immunoblotting. After transfection and treatment with cotransin (17 h, 30 μM) (+) or with DMSO (-) secretory and integral membrane proteins were isolated from HepG2 cells. The proteins were identified by SDS-PAGE/immunoblotting using specific primary antibodies and horseradish peroxidise-conjugated anti-mouse or anti-rabbit IgG as secondary antibodies. The cytosolic GAPDH protein does not contain a signal sequence and served as a control for a non-sensitive protein. As examples for secretory proteins (all SPs) cotransin-sensitive Apo B-100 and cotransin-resistant PAI-1 were used. For membrane protein possessing SPs, cotransin-sensitive CDH2 and cotransin-resistant CNX were analyzed. As examples for membrane proteins containing SASs, cotransin-sensitive Erlin2 and cotransin-resistant CLDN1 are shown. The immunoblots are representative of three independent experiments. The bar graphs shown at the right side of each immunoblot represent mean intensities of the respective protein bands of these three independent experiments ±SD (densitometric analysis using ImageJ).

To confirm the results of the SILAC experiments, expression of selected secretory or integral membrane proteins was analysed in cotransin-treated (30 μM) or DMSO-treated HepG2 cells by SDS-PAGE/immunoblotting with specific antibodies for the target proteins (Fig. 2). Secreted proteins were precipitated from the cell culture medium by TCA. For the detection of the membrane proteins, crude membrane fractions were used following cell fractionation. The protein glyceraldehyde-3-phosphate dehydrogenase (GAPDH) served as a control for a non-sensitive protein since this cytosolic protein does not contain a signal sequence. In the case of the secretory protein apolipoprotein B-100 (Apo B100) (SP) and the membrane proteins cadherin-2 (CDH2) (SP) and Erlin-2 (Erlin2) (SAS), expression was substantially decreased following cotransin treatment confirming the SILAC data (Fig. 2). The cotransin resistance of the secretory protein plasminogen activator inhibitor 1 (PAI-1) and the membrane proteins calnexin (CNX) (SP) and Claudin-1 (CLDN1) (SAS) could also be proved indicating that the generated dataset is reliable.

### Bioinformatic analysis of the SILAC dataset reveals a putative conformational consensus motif which may mediate cotransin sensitivity of SASs

To date, a consensus sequence mediating cotransin sensitivity of proteins is unknown. It is also not clear whether cotransin interacts with signal sequences directly in the protein-conducting channel or *via* an indirect mechanism. In the case of the SPs of VCAM1 and the vascular endothelial growth factor, amino acid residues were characterized which are responsible for the sensitivity to the HUN-7293 derivative CAM741 [9]. However, these SPs did not share sequence similarities and consequently a consensus sequence could not be defined [10]. We used the dataset of our SILAC study and bioinformatics tools to identify properties discriminating sensitive and non-sensitive signal sequences.

**Fig 3:**
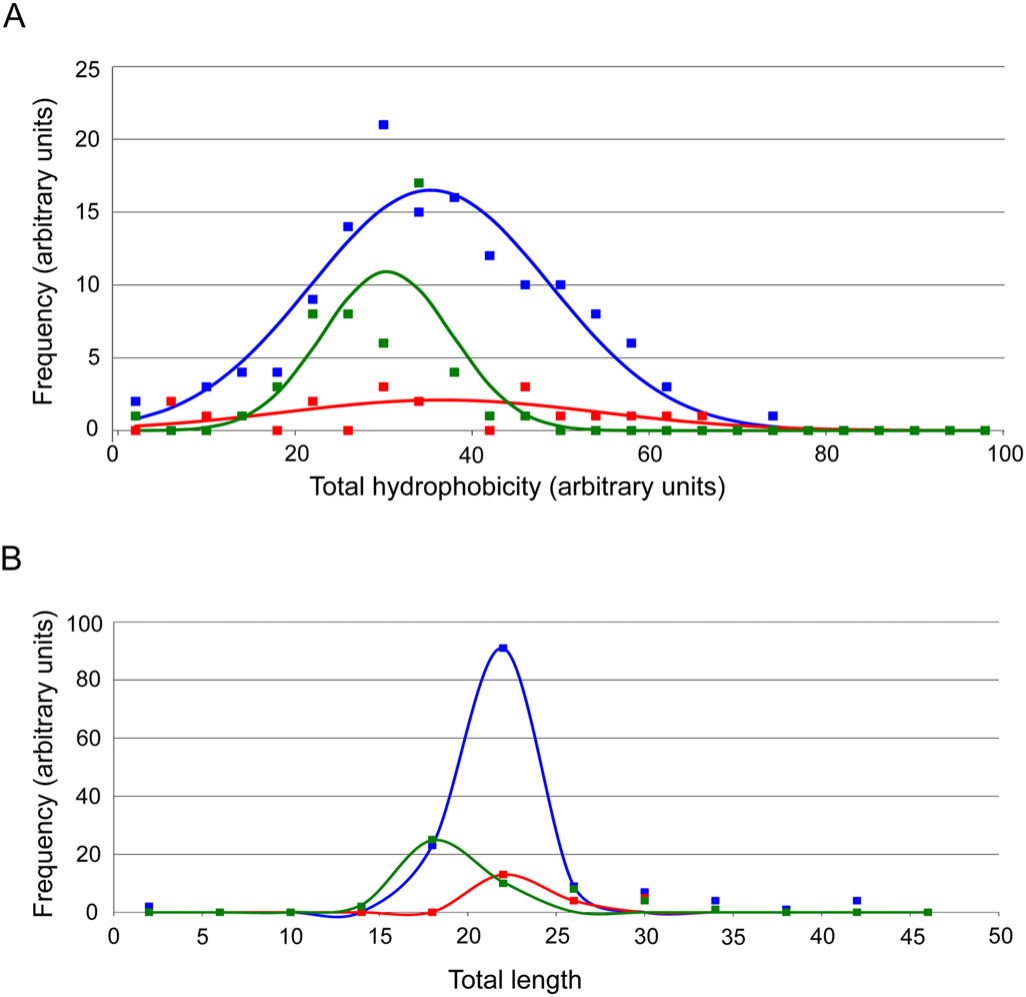
Bioinformatic analysis of the signal sequence properties of cotransin-sensitive and non-sensitive proteins. The blue, red and green fitted curves represent 143 non-ensitive integral membrane proteins, 21 sensitive integral membrane proteins and 50 sensitive secretory proteins respectively. **A**. Hydrophobicity analysis. The frequency of signal sequence hydrophobicity (sorted in classes of hydrophobicity ranging from 0–100 in steps of 4 in a scale of 0–25) was plotted against total hydrophobicity of the signal sequences (ranging from 0–100). **B**. Signal sequence length analysis. The frequency of signal sequence length (sorted in classes ranging from 0–50 amino acid residues in steps of 4 in a scale from 0–100) was plotted against total length of the signal sequences.

Secretory proteins possess invariantly SPs and the vast majority of those we identified were cotransin-sensitive. Since SPs are usually less hydrophobic than SASs, cotransin sensitivity may correlate with signal sequence hydrophobicity. However, such a correlation could not be found (Fig. 3A). In fact, hydrophobicity is rather variable among the sensitive SPs of secretory proteins and among sensitive and non-sensitive signal sequences of membrane proteins. Another possibility is that cotransin sensitivity may be related to signal sequence length. Likewise, it was not possible to obtain such a correlation either (Fig. 3B).

**Fig 4:**
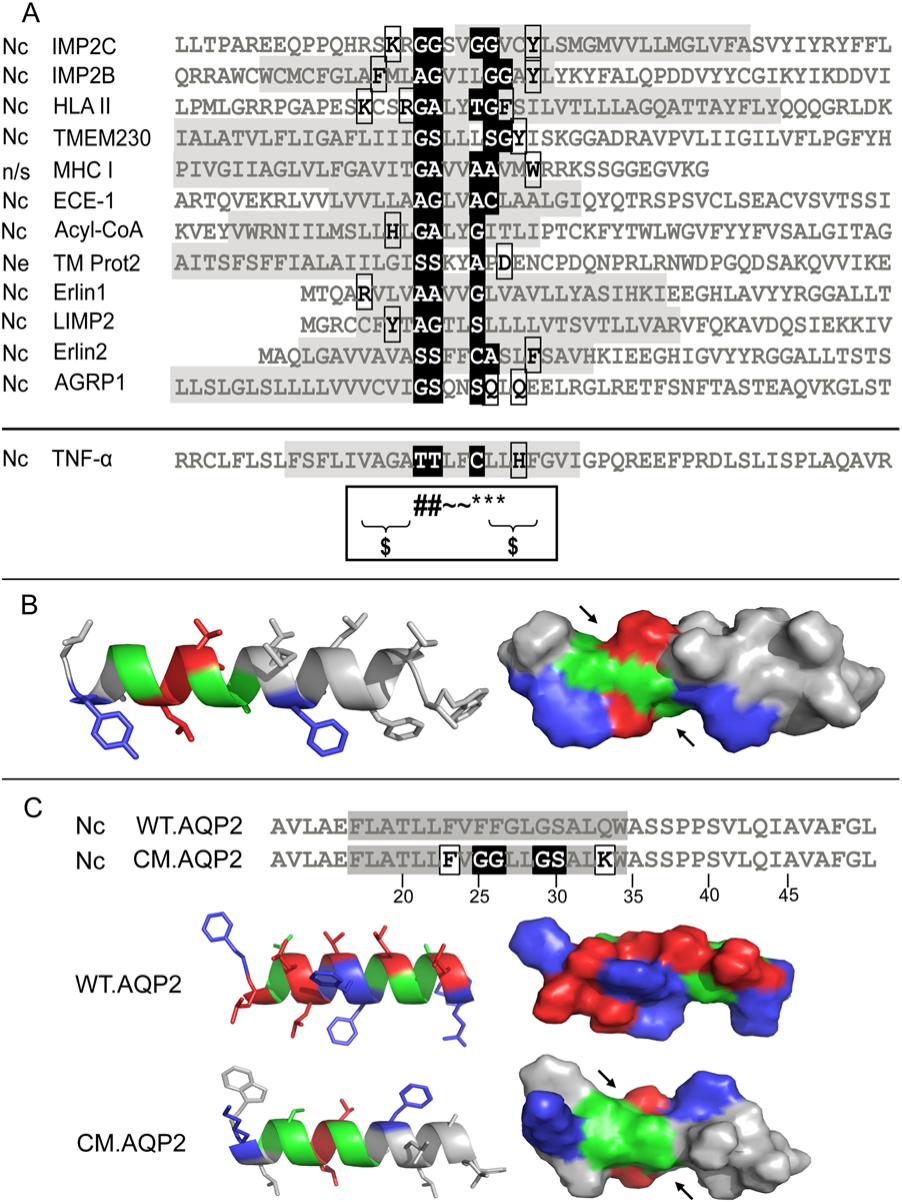
Identification of a putative conformational consensus motif in sensitive SASs. A. Sequence alignment. The sequences of the 12 sensitive SASs, identified in this study, are shown in grey (TM1 of the proteins). Cotransin sensitivity decreases from top to bottom. The sequence of the SAS of the TNF-α, which was previously shown to be cotransin-sensitive [8] is shown separately. Nc indicates a SAS with an N-terminus oriented towards the cytoplasm, Ne indicates a sequence with an N-terminus facing the extracellular site (ER: luminal side); n/s indicates an as yet non-specified N tail orientation. The consensus motif consists of two groups of small amino acids in the central part (black), which are separated by two or three bulky amino acid residues and flanked by large charged, large polar or aromatic amino acids residues (white with black frame). The black box below the alignment assigns the possible positions of the amino acid residues in the motif: (#) and (*) indicate the positions of the small amino acid residues forming the first and second cavities respectively; (~) and ($) indicate the amino acid residues separating and flanking the small residues respectively. The abbreviations are: IMP2C, integral membrane protein 2 C; IMP2B, integral membrane protein 2 B; HLA II, HLA class II histocompatibility antigen gamma chain; TMEM230, transmembrane protein 230; MHC I, MHC class I antigen; ECE-1, endothelin-converting enzyme 1; Acyl-CoA, Acyl-CoA desaturase; TM Prot2, transmembrane protein 2; Erlin1, Erlin-1; LIMP2, lysosome membrane protein 2; Erlin2, Erlin-2; AGPR1, asialoglycoprotein receptor 1; TNF-α, tumor necrosis factor-α. **B**. Exemplary α-helical (left) and solvent-reachable surface projection (right) of the SAS of IMP2B. Green colour represents the small amino acid residues forming the two cavities, red colour the separating residues and blue colour the flanking residues. The two cavities are indicated by arrows. **C**. Introduction of the identified conformational consensus motif into the SAS of the cotransin-resistant AQP2 protein. Upper panel: Alignment of the SAS (highlighted in grey) of construct WT.AQP2 and mutant CM.AQP2. In the case of CM.AQP2, the motif consists of the flanking amino acid residues (white with black frame), the small residues forming the cavities in the surface of the helix (black) and the separating more bulky residues lying between the cavities. Lower panel: α-helical structure of WT.AQP2 and mutant CM.AQP2 visualized using the program PyMol (left side). The structural level is shown by the solvent reachable surface of the α-helices (right side). Green colour represents the small amino acid residues forming the two cavities, red colour the separating residues and blue colour the flanking residues. The two cavities, which are a result of the mutations, are indicated by black arrows.

We next aligned all available sensitive and non-sensitive signal sequences to look for a consensus motif which may be associated with cotransin sensitivity. We failed to detect such a sequence for sensitive SPs in agreement with previous results [9, 10]. However, it was possible to derive a putative conformational consensus motif in the case of all 12 sensitive SASs of integral membrane proteins (Fig. 4A; cytosolic N tail: 10 sequences; extracellular N tail: 1 sequence; N tail orientation not specified: 1 sequence). The central part of this motif contains two patches of small amino acid residues (Gly, Ala, Ser or Thr, Cys), the first formed by two and the second either by one or two residues. These two patches are separated by two or three bulky amino acid residues and flanked on one or both sides either by large polar, charged or aromatic residues (Fig. 4A). Assuming a strict α-helical structure of the SAS, the structural consequence of this motif is the formation of two distinct cavities formed by the small amino acid residues in the surface of the helical structure (Fig. 4B). In the case of the non-sensitive membrane proteins, the motif was found only in 5 out of 143 sequences demonstrating a highly significant correlation between the presence of the motif in an SAS and its cotransin sensitivity (p value < 0.0001 according to Fisher´s exact test) [21]. The motif is also present in the SAS of the TNF-α which was previously reported as cotransin-sensitive [8] (Fig. 4A, last sequence in the alignment). As mentioned above, the motif is not present in SPs. None of the 50 sensitive SPs of secretory proteins and only 2 of the sensitive SPs of integral membrane proteins carried a similar sequence.

### Experimental confirmation that the putative conformational consensus motif mediates cotransin sensitivity of SASs

**Fig 5:**
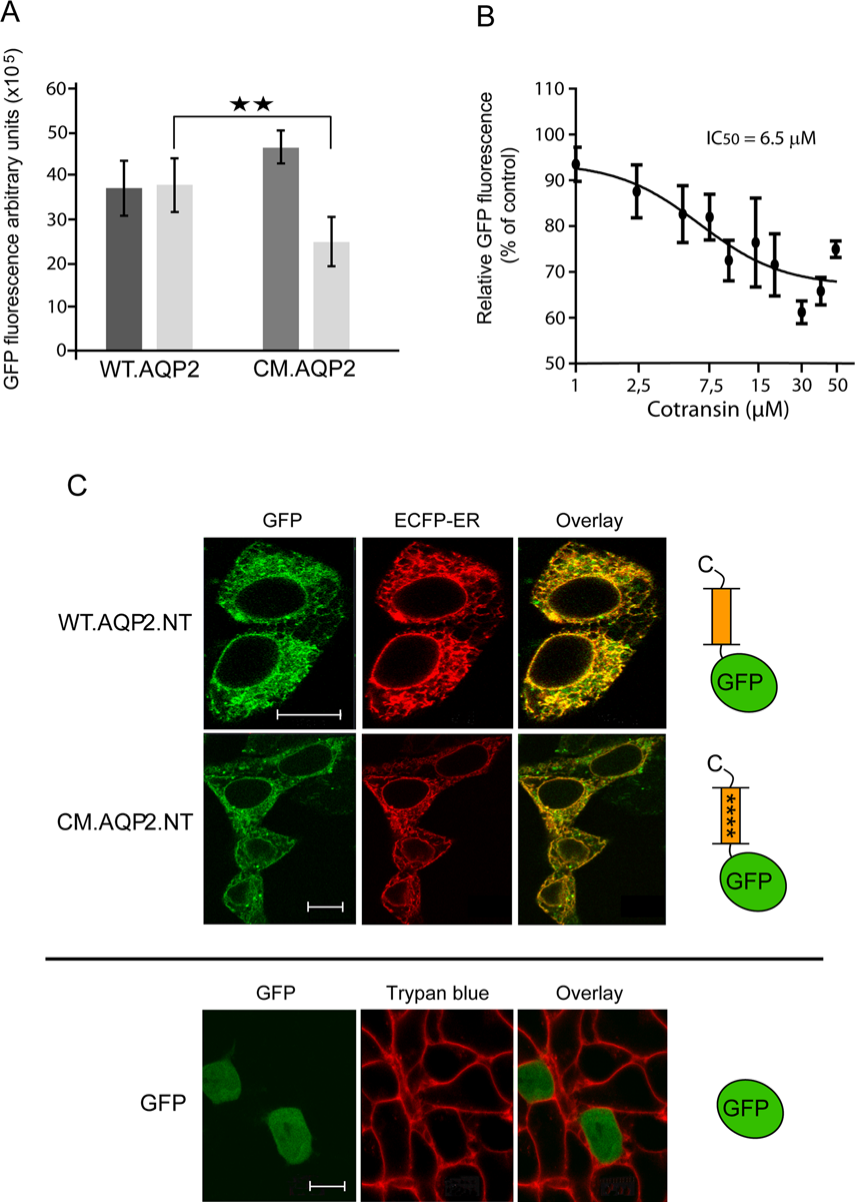
Experimental validation of the introduced consensus motif in transiently transfected HEK 293 cells. A. Biosynthesis of constructs WT.AQP2 and CM.AQP2 following cotransin treatment. Columns represent the GFP fluorescence signals (arbitrary units) of the constructs after 19 h of treatment with 1.5% DMSO solvent alone (-) (dark grey columns) or 10 μM cotransin/1.5% DMSO (+) (light grey columns). Shown are mean values of three independent experiments ± SD. Fluorescence was quantified by flow cytometry measurements. **B**. Concentration-response curve for the cotransin-mediated biosynthesis inhibition of CM.AQP2. Data points represent the mean values of the GFP fluorescence signals of the construct after 19 h of treatment with increasing concentrations of cotransin (1–50 μM in 1.5% DMSO). Shown are mean values of three independent experiments ± SD. Fluorescence was quantified by flow cytometry measurements as above and data were normalized to the DMSO control (1.5% DMSO). The calculated IC_50_ value is indicated. **C**. Upper Panel. Colocalization of the GFP fluorescence signals of the truncated constructs WT.AQP2.NT and CM.AQP2.NT (left side, green) with the CFP fluorescence signals of the cotransfected ER marker ECFP-ER (middle, red). The fluorescence signals were recorded using confocal LSM and computer-overlayed (right side; colocalization is indicated by yellow color). The xy-scans show representative cells and are representative of three independent experiments. The cartoon on the right side shows a schematic depiction of the constructs. The 4 point mutations of construct CM.AQP2.NT are indicated by (****). Lower panel. Subcellular localization of unfused soluble GFP (control protein which does not contain a signal sequence). For clarity, the GFP fluorescence signals (left side, green) and those of the plasma membrane dye trypan blue (middle, red) were recorded in this case and computer overlayed (right panel). The xy-scans show representative cells and are representative of three independent experiments.

To demonstrate that the identified sequence is indeed responsible for the cotransin sensitivity of SASs, we introduced the motif into a cotransin resistant SAS. We took the aquaporin 2 (AQP2) water channel protein as a model, a hexahelical integral membrane protein with cytosolic N and C tail. To introduce the two cavities of the motif, the point mutations F25G, F26G, G27L, Q33K were introduced into the TM1 of a C-terminally GFP-tagged variant of AQP2 (resulting constructs: mutant F25G, F26G, G27L, Q33K = CM.AQP2; wild type = WT.AQP2). The structural consequences of these mutations are shown in Fig. 4C; the resulting two cavities are indicated by black arrows. HEK 293 cells were transiently transfected with the constructs and treated with cotransin (10 μM) or DMSO solvent or cycloheximide (0.1μg/ml). After 19 h of incubation, the total GFP fluorescence of 1 x 10^4^ cells was analyzed using flow cytometry as a measure of biosynthesis. To avoid falsification of the results by proteins already synthesized at t_0_ of cotransin treatment, cycloheximide values were subtracted. In the case of mutant CM.AQP2, a significant reduction of the GFP fluorescence signals was observed indicating that introduction of the consensus sequence by the 4 point mutations indeed induced cotransin sensitivity of the SAS (Fig. 5A). Using the same flow cytometry assay but variable cotransin concentrations, a concentration-response curve could be derived for the cotransin-mediated biosynthesis inhibition of CM.AQP2 (Fig. 5B). The calculated IC_50_ value of 6.5 μM is comparable to those described previously (e.g. endothelin B receptor = 5.4 μM; reference 6).

However, under careful consideration, it can not be excluded that the mutations led to a non-functional signal anchor sequence of AQP2 in the above experiment. In this case, one of the more C-terminal located transmembrane domains could function as an alternative SAS [22] which may possess the observed higher cotransin sensitivity in comparison to the wild type TM1. To rule out this possibility, truncated variants of the above proteins were constructed encoding only the N tail, TM1 and the first extracellular loop of AQP2. Both constructs were N-terminally tagged with GFP (resulting constructs: mutant F25G, F26G, G27L, Q33K = CM.AQP2.NT; wild type = WT.AQP2.NT). In these fusions, TM1 is the sole transmembrane domain which can function as a SAS. To analyze targeting of the constructs to the ER membrane we used a previously published microscopical assay which is based on the localization of the fluorescence signals of GFP fusion proteins [23, 24]. If a fused sequence could function either as a SP or a SAS, the GFP moiety is targeted to the ER membrane leading to a reticular GFP fluorescence pattern typical for the ER. Under these conditions, the nucleus is free of GFP fluorescence. If a fused sequence was unable to function as a signal sequence, GFP is located in the cytosol leading to a diffuse fluorescence pattern filling the cell’s interior. Moreover, due to the nuclear targeting signal of GFP [25] fluorescence signals are also detectable in the nucleus. HEK 293 cells were transiently co-transfected with the constructs and the ER marker ECFP-ER and colocalization of the GFP fluorescence signals with the signals of ECFP-ER was analyzed using LSM (Fig. 5C, upper panel). Both WT.AQP2.NT and CM.AQP2.NT showed a reticular fluorescence pattern and could be readily colocalized with the ER marker. We also used unfused soluble GFP as a control for a protein which does not contain a SAS. In contrast to WT.AQP2.NT and CM.AQP2.NT, this protein was distributed diffusely throughout the cells and was also transferred to the nucleus (Fig. 5C, lower panel). These data show that the SAS of CM.AQP2.NT is still functional, despite the mutations. Taken together, these results demonstrate that the identified putative conformational consensus motif of SASs is indeed involved in mediating cotransin sensitivity.

## Discussion

In the previous studies for cotransin and the related cyclodepsipeptides [5, 6, 8–10] only a small subset of sensitive proteins was identified. In particular proteins with lower sensitivity were not studied systematically and not differentiated from completely resistant proteins. Moreover, it was unknown whether SASs may be affected by cotransin in significant amounts. A consensus motif in signal sequences mediating cotransin sensitivity could also not be defined. We performed a proteomic SILAC approach on HepG2 cells using saturating cotransin concentrations (30 μM) to address these questions. Under these conditions, almost all identified secretory proteins were cotransin-sensitive whereas the majority of the integral membrane proteins were resistant. Given the fact that the SILAC experiments failed to detect expression of very lowly expressed proteins and that HepG2 cells do not express the complete proteome, it is conceivable that the number of the actual cotransin-sensitive proteins is still underestimated. The idea to develop cotransin analogues affecting the synthesis of individual proteins, i.e. to get from a selective to a specific substance by derivatization [6], will thus be difficult to achieve.

In our study, only two secretory proteins were found to be completely cotransin-resistant, namely the plasminogen activator inhibitor 1 and calumenin [26–29]. Although both proteins contain SPs according to the UniProt data base [30–32], it should be analyzed whether these proteins may use a secretion pathway independent of Sec61 such as the recently described Sec62 pathway [33]. Interestingly, the expression of one protein, namely the placental protein 12 [34] seems to be up-regulated following cotransin treatment. In this particular case, cotransin may strengthen the interaction of the putative SP with Sec61. More likely, however, the substance could inhibit the biosynthesis of an unknown protein involved in down-regulation of placental protein 12.

Our results for integral membrane proteins rule out the possibility that cotransin might affect exclusively SPs of membrane proteins since a significant amount of SASs was inhibited, too. Moreover, in the case of sensitive integral membrane proteins, the data revealed that both types of signal sequences seem to be affected without preference.

Using bioinformatic tools, we could prove that cotransin sensitivity of a signal sequence neither correlates with signal sequence length nor with its hydrophobicity. Moreover, a general consensus motif mediating cotransin sensitivity, which is present in both SPs and SASs, does obviously not exist. In the case of SPs, the failure to define a consensus sequence is in agreement with previous results [9, 10]. In contrast to the situation with SPs, however, we were able to define a conformational consensus motif in sensitive SASs. By introduction of the motif into the cotransin-resistant SAS of AQP2, the functionality of this motif could be confirmed. In a recent study for TNF-α, residues T45 and T46 of its SAS were shown to determine cotransin sensitivity [7]. These findings are easily explicable by our data: residues T45/T46 build the first cavity of the conformational consensus motif which is also present in the SAS of TNF-α (Fig. 4A, last sequence in the alignment, first black-shaded letters).

Taken together, our results may lead to a modified (but still highly speculative) working hypothesis for the mechanism of action and selectivity of cotransin. As suggested previously, cotransin may replace sensitive signal sequences from their acceptor sites in Sec61 [5, 9]. The fact that the motif was only detectable in SASs but not in SPs suggests that there may be (at least slightly) different binding sites for signal sequences in Sec61 with variable responsiveness to the compound. The majority of the SASs are cotransin-resistant and these sequences may interact with contact sites which are not accessible for the substance. Sensitive SASs may bind to an alternative site which can be influenced by the compound. Binding behaviour of these sensitive SASs may then be determined by the identified conformational consensus motif. The various SPs could again interact with a slightly different site which is in principle cotransin-sensitive but whose responsiveness is more distinctly dependent on cotransin concentrations.

Whereas the characterization of a sequence motif mediating cotransin sensitivity of a subgroup of proteins represents a step forward, the mechanism of action and selectivity of cotransin is far away from a complete understanding. Most progress would come, of course, from the identification of the binding sites of cotransin itself.

## Supporting Information

S1 Table Cotransin-sensitive and non-sensitive secretory and integral membrane proteins detected by SILAC and quantitative mass spectrometry. The UniProt identification number, ratio of the forward and backward experiment, signal sequence type (Sp or SAS) and signal sequence length are indicated.
(PDF)
