# Supplementary material for: Defining a Conformational Consensus Motif in Cotransin-Sensitive Signal Sequences: A Proteomic and Site-Directed Mutagenesis Study
Source: PLoS One. 2015 Mar 25;10(3):e0120886. doi: 10.1371/journal.pone.0120886 (PMC4373898; doi:10.1371/journal.pone.0120886)
Supplement: S1 Table — The UniProt identification number, ratio of the forward and backward experiment, signal sequence type (Sp or SAS) and signal sequence length are indicated. (PDF) [file pone.0120886.s001.pdf]

S1 Table. Cotransin-sensitive and non-sensitive secretory and integral membrane proteins detected by SILAC and quantitative mass spectrometry.

The UniProt identification number, ratio of the forward and backward experiment, signal sequence type (Sp or SAS) and signal sequence length are indicated.

Sensitive integral membrane proteins					
Name of the protein	# UniProt.	Ratio H/L for	Ratio H/L rev	Signal sequence	# of AA
Integral membrane protein 2C	Q9NQX7-1	20,746	10,168	SAS	21
Integral membrane protein 2B	Q9Y287	12,227	5,0837	SAS	21
HLA class II histocompatibility antigen gamma chain	P04233-1	10,474	9,6608	SAS	26
Trans-Golgi network integral membrane protein 2	O43493-1	4,696	4,4835	SP	21
Transmembrane protein 230	Q96A57-2	4,1618	4,0131	SAS	21
MHC class I antigen	A5I8L1	3,9012	3,4994	not specified	27
Endothelin-converting enzyme 1	P42892-1	3,8418	3,2381	SAS	21
Acyl-CoA desaturase	O00767	3,7245	5,0245	SAS	22
HLA class I histocompatibility antigen, A-24 alpha chain	P05534	3,2443	4,2437	SP	24
Procollagen-lysine, 2-oxoglutarate 5-dioxygenase 2	O00469-2	2,9751	3,4644	SP	25
Neurotensin receptor 3	Q99523	2,3567	1,9949	SP	33
Integrin alpha-6	P23229-1	2,1745	2,5061	SP	23
Carboxypeptidase D	O75976	2,1539	2,0951	SP	31
Nodal modulator 2	Q5JPE7-1	1,975	2,2119	SP	31
Transmembrane protein 2	Q9UHN6	1,9053	2,3061	SAS	21
Amyloid-like protein 2	Q06481-1	1,8713	1,824	SP	31
Erlin-1	O75477	1,8526	1,7503	SAS	21
Lysosome membrane protein 2	Q14108	1,8332	1,8929	SAS	23
Erlin-2	O94905-1	1,8324	1,6993	SAS	21
Cadherin-2	P19022	1,7095	1,8931	SP	25
Asialoglycoprotein receptor 1	P07306	1,7332	1,6847	SAS	21

Insensitive integral membrane proteins					
Name of the protein	# UniProt	Ratio H/L for	Ratio H/L rev	Signal sequence	# of AA
MAN1A1 protein	Q6P052	2,7095	1,2161	SAS	21
Zinc transporter ZIP14	Q15043-1	1,9032	1,35	SP	30
Syndecan-2	P34741	1,7551	1,4943	SP	18
Tyrosine-protein kinase-like 7	Q13308	1,7351	1,441	SP	30
Lutheran blood group glycoprotein	P50895	1,6274	1,9938	SP	31
Melanoma inhibitory activity protein 3	Q5JRA6-1	1,6354	1,656	SP	22
Putative uncharacterized protein KIAA0090	Q8N766-1	1,6106	1,9692	SP	21
Cysteine-rich fibroblast growth factor receptor	Q6P9D1	1,5577	1,7971	SP	29
Malectin	Q14165	1,509	1,6275	SP	28
Aminopeptidase N	P15144	1,5021	1,6839	SAS	24
Immunoglobulin superfamily member 1	Q8N6C5-1	1,4825	1,8491	SP	28
Protein-tyrosine phosphatase-like member B	Q6Y1H2	1,4814	1,3652	SAS	19
Vesicular integral-membrane protein VIP36	Q12907	1,4737	1,4328	SP	44
Nicastrin	Q92542-1	1,461	1,4247	SP	33
Inhibitor of nuclear factor kappa-B kinase-interacting protein	Q70UQ0-4	1,4519	1,2783	SAS	17
Protein sel-1 homolog 1	Q9UBV2-1	1,4318	1,2956	SP	21
Lysosome-associated membrane glycoprotein 1	P11279	1,3968	1,3662	SP	28
Synaptogyrin-2	Q3KQZ2	1,3905	1,2768	SAS	19
Vesicle-associated membrane protein 8	Q9BV40	1,3629	1,0483	SAS	21
Tyrosine-protein kinase receptor ECK	B4DL04	1,3612	1,7539	SAS	22
Integrin alpha-2	P17301	1,3412	1,8663	SP	29
Secretory carrier-associated membrane protein 2	O15127	1,327	1,2852	SP	21
Zinc transporter SLC39A7	Q92504	1,3251	1,1509	SAS	21
Activated leukocyte cell adhesion molecule	Q13740-1	1,3225	1,1838	SP	27
Transmembrane emp24 domain-containing protein 5	Q9Y3A6	1,322	1,2993	SAS	21
Signal peptidase complex catalytic subunit SEC11A	P67812	1,3198	1,3276	SAS	20
Scavenger receptor class B member 1	Q8WTV0-1	1,3198	1,4335	SAS	21
Integrin beta-1	Q8WUM6	1,3187	1,3434	SP	20
Transmembrane protein 4	Q9Y2B0-1	1,2986	1,3775	SP	20
Receptor expression-enhancing protein 5	Q00765	1,2928	1,0779	SAS	21
Synaptobrevin-3	A8MVP3	1,2725	1,0188	SAS	20
Endoplasmic reticulum stress-response protein 25	Q7Z7H5-1	1,2725	1,2331	SP	29
Transmembrane emp24 domain-containing protein 9	Q9BVK6	1,2716	1,2101	SP	35
Syntaxin-7	O15400-1	1,2711	1,2194	SAS	21
Na(+)/K(+) ATPase alpha-1 subunit	P05023-1	1,2554	1,4522	SAS	21
Transmembrane protein Tmp21	P49755	1,2511	1,3358	SP	31
Putative uncharacterized protein KIAA2013	Q8IYS2-2	1,2511	1,7214	SP	40
Nck-associated protein 1	Q9Y2A7	1,2502	0,83148	SAS	21
Dolichyl-diphosphooligosaccharide-protein glycosyltransfera	P39656	1,2485	1,2281	SP	42
ER-Golgi intermediate compartment 32 kDa protein	Q969X5-1;Q	1,2447	1,1686	SAS	21
Lectin mannose-binding 2-like	Q9H0V9-2	1,2357	1,4471	SP	44
Probable ergosterol biosynthetic protein 28	Q9UKR5	1,2325	1,1965	SAS	21
Signal peptidase complex subunit 1	Q9Y6A9	1,2287	1,3074	SAS	21

Signal recognition particle receptor subunit alpha	P08240	1,2269	1,0346	SP	18
Signal sequence receptor subunit delta	P51571	1,2229	1,2164	SP	23
Receptor expression-enhancing protein 6	Q96HR9	1,2186	1,2695	SAS	21
Sterol-4-alpha-carboxylate 3-dehydrogenase, decarboxylatin	Q15738	1,207	1,1602	SAS	21
Lanosterol 14-alpha demethylase	Q16850	1,1934	1,7442	SAS	21
ER-Golgi intermediate compartment 53 kDa protein	P49257	1,1829	1,1029	SP	30
HIG1 domain family member 2A	Q9BW72	1,1779	1,1289	SAS	21
Interleukin-1 receptor-like 1 ligand	Q13445	1,176	1,3355	SP	23
UPF0389 protein FAM162A;E2-induced gene 5 protein	Q96A26	1,1735	1,0285	SAS	18
Junctional adhesion molecule 1	Q9Y624	1,1724	1,3617	SP	27
Signal peptidase complex subunit 2	Q15005	1,1706	1,0902	SAS	21
Dolichyl-diphosphooligosaccharide-protein glycosyltransfera	P04844	1,1683	1,3029	SP	22
Leukocyte activation antigen M6	P35613-1	1,1662	1,3153	SP	21
Signal sequence receptor subunit alpha	P43307-1	1,1649	1,2662	SP	18
Syntaxin 12	B1AJQ6	1,1637	1,3966	not specified	0
Fibronectin type III domain-containing protein 3B	Q53EP0-1	1,1587	1,1155	SAS	21
Signal peptidase complex subunit 3	P61009	1,1566	1,3295	SAS	21
Farnesyl-diphosphate farnesyltransferase	P37268	1,1512	1,0264	SAS	21
Flotillin-1	O75955	1,1502	1,3594	not specified	0
Thioredoxin-related transmembrane protein 1	Q9H3N1	1,1487	1,3325	SP	26
Transmembrane emp24 domain-containing protein 2	Q15363	1,1464	1,3904	SP	20
Inhibitor of nuclear factor kappa-B kinase-interacting protein	Q70UQ0-1	1,1394	1,324	SAS	17
Toll-like receptor adapter molecule 2 (TICAM-2)	Q9Y3B3	1,1387	1,5627	SP	34
Transferrin receptor protein 1	P02786	1,1362	1,0995	SAS	21
Kinesin receptor	Q86UP2-1	1,1356	1,1007	SAS	23
Transmembrane protein 214	Q6NUQ4	1,133	1,1083	SAS	21
Alpha-mannosidase 2	Q16706	1,1322	1,25	SAS	21
Sodium/potassium-transporting ATPase subunit beta-1	P05026-1	1,1265	1,3039	SAS	28
Desmoglein-2	Q14126	1,1257	1,3724	SP	23
ORM1-like protein 1	Q8N138-1	1,1238	1,3964	SAS	21
Aspartyl/asparaginyl beta-hydroxylase	Q12797	1,123	1,1982	SAS	21
Signal sequence receptor subunit gamma	Q9UNL2	1,1219	1,1119	SAS	21
Sphingosine-1-phosphate lyase 1	O95470	1,1218	1,1105	SAS	21
Oxidoreductin-1-L-alpha	Q96HE7	1,1175	1,3324	SP	23
Cytoskeleton-associated protein 4	Q07065-1	1,1035	1,1357	SAS	21
Cell adhesion molecule 1	Q9BY67-2	1,0866	1,142	SP	44
Coiled-coil domain-containing protein 47	Q96A33-1	1,0846	1,1767	SP	20
ATP-binding cassette sub-family D member 3	P28288-1	1,0824	1,0306	SAS	21
Syntaxin-4	Q12846	1,0804	1,0392	SAS	21
Dolichyl-diphosphooligosaccharide-protein glycosyltransfera	P04843	1,076	1,1044	SP	23
Mannosyl-oligosaccharide glucosidase	Q13724	1,075	1,207	SAS	21
Retinol dehydrogenase 11	Q8TC12-1	1,0747	1,1386	SAS	21
Transmembrane and coiled-coil domain-containing protein 1	Q9UM00-1	1,0738	1,2422	SAS	21
Serine palmitoyltransferase 1	O15269	1,0731	1,1363	SAS	21
Dolichyl-diphosphooligosaccharide-protein glycosyltransfera	P46977	1,0709	1,2231	SAS	21
Plexin-B2	O15031	1,0689	1,1718	SP	19
Lymphocyte activation antigen 4F2 large subunit	P08195	1,0663	1,1863	SAS	21
Monocarboxylate transporter 1	P53985	1,0631	1,307	SAS	21
Transmembrane protein 111	Q9P0I2-1	1,0625	1,1518	SAS	21
Coiled-coil domain-containing protein 56	Q9Y2R0	1,0576	0,93162	SAS	21
Major histocompatibility complex class I antigen-binding prot	P27824	1,051	1,1415	SP	20
Vesicle-trafficking protein SEC22b	O75396	1,0503	1,0613	SP	21
Alkaline phosphodiesterase I	P22413	1,0465	1,6024	SAS	21
Erythrocyte band 7 integral membrane protein	P27105	1,0385	1,1778	SAS	29
Transmembrane protein 97	Q5BJF2	1,0384	1,338	SAS	21
B-cell receptor-associated protein 31	P51572	1,0365	1,0912	SAS	21
Synaptic glycoprotein SC2	Q9NZ01-1	1,0309	1,1084	SAS	21
Vesicle-associated membrane protein-associated protein B/(	O95292-1	1,0282	1,0623	SAS	21
Dolichyl-phosphate beta-glucosyltransferase	Q9Y673	1,0238	1,0508	SAS	21
Reticulon-3	O95197-1	1,0212	0,90181	SAS	21
Microsomal glutathione S-transferase 1	P10620	1,0202	0,87959	SAS	24
Estradiol 17-beta-dehydrogenase 12	Q53GQ0	1,0157	1,0915	SAS	21
Transmembrane 9 superfamily member 4	Q92544-1	1,0101	1,3634	SP	23
Neutral amino acid transporter B(0)	Q15758	1,0086	1,2055	SAS	21
Protein jagunal homolog 1	Q8N5M9	1,0002	1,0546	SAS	21
Claudin-1	O95832	0,99672	0,99112	SAS	21
CAAX prenyl protease 1 homolog	O75844	0,99662	0,98685	SAS	21
Cytochrome b5	P00167-1	0,99497	0,99787	SAS	23
Dipeptidyl peptidase 4	P27487	0,99432	1,1831	SAS	22
Fatty aldehyde dehydrogenase	P51648-2	0,98678	1,0054	SAS	17
Prostaglandin E synthase 2	Q9H7Z7	0,96913	1,0138	SAS	17
Membrane-associated progesterone receptor component 1	O00264	0,95886	1,0866	SAS	19
Long-chain-fatty-acid-CoA ligase 4	O60488-1	0,957	0,84243	SAS	21
Extended synaptotagmin-2	A0FGR8-1	0,95423	1,0782	SAS	21
Protein tyrosine phosphatase-like protein PTPLAD1	Q9P035	0,95001	1,124	SAS	21
Epoxide hydrolase 1	P07099	0,94965	1,1234	SAS	19
Leucine-rich repeat-containing protein 59	Q96AG4	0,94092	0,94498	SAS	21

Intercellular adhesion molecule 1	P05362	0,94001	1,3436	SP	27
Transmembrane protein 33	P57088	0,92658	1,0075	SAS	21
p180/ribosome receptor	A7BI36	0,91736	0,97114	SAS	18
Extended synaptotagmin-1	Q9BSJ8-1	0,91598	0,94843	SAS	21
24-dehydrocholesterol reductase	Q15392	0,90654	0,97957	SP	22
Sarcoplasmic/endoplasmic reticulum calcium ATPase 2	P16615-1	0,89186	0,92792	SAS	21
Biliary glycoprotein	Q13857	0,89167	1,1469	SP	34
Surfeit locus protein 4	O15260-1	0,86779	0,86794	SAS	21
Membrane-associated progesterone receptor component 2	O15173	0,86626	1,0411	SAS	25
Monocarboxylate transporter 4	O15427	0,85767	1,067	SAS	21
Long-chain-fatty-acid--CoA ligase 1	P33121-1	0,84408	0,8604	SAS	21
CDGSH iron sulfur domain-containing protein 2	Q8N5K1	0,84044	0,94488	SAS	23
Golgin B1	B2ZZ91	0,82226	0,90211	SAS	19
Solute carrier family 2, facilitated glucose transporter membe	P11166	0,80711	0,99644	SAS	21
Adipocyte plasma membrane-associated protein	Q9HDC9	0,79224	1,2584	SAS	21
Protein transport protein Sec61 subunit beta	P60468	0,78201	0,64049	SAS	21
Translocation protein SEC62	Q99442	0,77787	1,0036	SAS	21
Alpha-1,3-mannosyltransferase ALG2	Q9H553-1	0,7427	0,6919	SAS	21
Signal recognition particle receptor subunit beta	Q9Y5M8	0,7397	0,81726	SAS	21
Translocation protein SEC63 homolog	Q9UGP8	0,72305	0,75133	SAS	21
Solute carrier family 2, facilitated glucose transporter membe	P11169	0,68328	0,8265	SAS	21
Protein transport protein Sec61 subunit alpha isoform 1	P61619-1	0,63756	0,68204	SAS	20
Minor histocompatibility antigen H13	Q8TCT9-1	0,63521	0,81577	SAS	21

Sensitive secretory proteins				
Name of the protein	# UniProt	Ratio H/L for	Ratio H/L rev	# of AA
Carboxypeptidase E	P16870	103,21	58,579	25
Apolipoprotein E	P02649	60,761	49,176	18
Apolipoprotein M	O95445	49,997	39,308	22
Bactericidal/permeability-increasing protein-like 1	Q8N4F0	75,505	38,078	20
Alpha-2-antiplasmin	Q8N5U7	42,837	35,891	27
Alpha-fetoprotein	P02771	68,33	34,42	18
Alpha-2-HS-glycoprotein	P02765	38,907	26,149	18
Vitronectin	P04004	4,5056	25,94	19
Inter-alpha-trypsin inhibitor heavy chain H2	P19823	35,258	24,287	18
cDNA, FLJ92861	B2R6A4	43,042	20,586	19
Antithrombin-III	P01008	29,224	19,32	32
Carboxypeptidase N subunit 2	P22792	29,709	19,098	21
Complement factor B	P00751-1	30,38	19,069	25
Inter-alpha-trypsin inhibitor heavy chain H3	Q06033-1	36,376	18,588	20
Lysozyme C	P61626	24,219	18,2	18
Complement factor I	P05156	29,844	18,063	18
Alpha-1-antichymotrypsin	P01011-2	28,2	17,207	23
Gastricsin	P20142	32,052	16,873	16
Apolipoprotein A-I	P02647	25,833	16,364	18
Alpha-2-macroglobulin	P01023	21,083	16,046	23
Complement C3	P01024	21,826	16,041	22
Granulins	P28799-1	16,098	15,829	17
Neuroserpin	Q99574	39,699	15,699	16
Apolipoprotein B-100	P04114	28,098	15,221	27
Growth/differentiation factor 15	Q99988	18,846	13,818	29
Thyroxine-binding globulin	P05543	22,912	13,635	20
Plasma serine protease inhibitor	P05154	25,154	13,004	19
Corticosteroid-binding globulin	P08185	41,117	12,813	22
Alpha-1-acid glycoprotein 1	P02763	22,819	12,396	18
Alpha-1-acid glycoprotein 2	P19652	23,063	12,345	18
Phospholipid transfer protein	P55058-1	7,8914	12,183	17
Semaphorin-3B	Q13214-1	22,9	12,056	24
Vitamin K-dependent protein S	P07225	18,113	11,877	24
Haptoglobin	P00738	18,881	10,292	18
Ceruloplasmin	P00450	7,696	7,8334	19
Proprotein convertase subtilisin/kexin type 9	Q8NBP7-1	21,324	7,7439	30
Cystatin-C	P01034	13,994	7,6667	26
Tissue factor pathway inhibitor	P10646-1	14,017	7,2468	28
C3/C5 convertase	P06681	12,501	7,2281	20
Leucine-rich alpha-2-glycoprotein	P02750	8,4702	7,1283	35
Plasma alpha-L-fucosidase	Q9BTY2	8,5838	7,0784	28
Protein AMBP	P02760	9,5837	6,8381	19
Ribonuclease T2	A6XND5	12,327	6,7854	27
Zinc-alpha-2-glycoprotein	P25311	10,148	6,1389	20
Fibulin 1	B0QY41	10,042	6,0488	29
Retinol-binding protein 4	P02753	8,701	4,7938	18
Serpin peptidase inhibitor, clade D (Heparin cofactor), meml	Q8IVC0	3,8316	4,3165	20
Alpha-1B-glycoprotein	A8K052	4,4223	4,0615	21
Insulin-like growth factor II	P01344-2	4,9995	3,0618	24
Kallistatin	P29622	13,655	2,8526	20

Insensitive secretory proteins				
Name of the protein	# UniProt	Ratio H/L for	Ratio H/L rev	# of AA
Calumenin	O43852-1	1,5003	1,4336	19
Plasminogen activator inhibitor 1	P05121	1,3761	1,053	23

Upregulated secretory proteins				
Name of the protein	# UniProt	Ratio H/L for	Ratio H/L rev	# of AA
Placental protein 12	P08833	0,10672	0,098323	25
